# A comparison of effectiveness of the contrast enhanced computed tomography with magnetic resonance imaging in the differential diagnosis of clear cell renal carcinoma

**DOI:** 10.2478/raon-2025-0033

**Published:** 2025-06-16

**Authors:** Tomasz Blachura, Julia Radzikowska, Patrycja S. Matusik, Aleksander Kowal, Jarosław D Jarczewski, Łukasz Skiba, Tadeusz J Popiela, Robert Chrzan

**Affiliations:** 1 Department of Diagnostic Imaging, University Hospital, Kraków, Poland; 2 Student’s Scientific Group, Jagiellonian University Medical College, Kraków, Poland; 3 Department of Radiology, Jagiellonian University Medical College, Kraków, Poland; 4 Department of Neurosurgery, Copernicus Memorial Hospital in Łódź, Comprehensive Cancer Centre and Traumatology, Łódź, Poland; 5 Department of Radiology, Ludwik Zamenhof University Children’s Clinical Hospital, Białystok, Poland

**Keywords:** RCC, renal masses, computed tomography, magnetic resonance imaging

## Abstract

**Background:**

The incidental detection of indeterminate small renal masses (SRMs) has been rising continuously over the last few decades. The aim of our study was to assess selected contrast enhanced computed tomography (CECT) parameters in the characterization of indeterminate SRMs and compare them with selected magnetic resonance imaging (MRI) data.

**Patients and methods:**

Patients with indeterminate SRMs discovered on CECT were included in the study. Selected CECT features have been analyzed as differentiating between clear cell renal cell carcinoma (ccRCC) and other etiologies of SRMs. In 82% of patients, which had available MRI data, a comparison between selected MRI and CECT parameters were performed.

**Results:**

Relative washout in CECT had the best accuracy (76.5%), sensitivity (88.9%), as well as satisfactory specificity (69.7%) in ccRCC prediction. The cut-off point determined in receiver operating analysis using the Youden index for this parameter was 11.54. Multivariable analysis showed that only T1 SI ratio < 0.73 from MRI parameters and relative washout > 11.5 from CECT parameters were independent predictors of ccRCC (OR: 30.86, 95% CI: 1.58-600.26, p = 0.024; OR: 15.36, 95% CI: 1.52-155.16, p = 0.021).

**Conclusions:**

In clinical practice, the use of both CECT and MRI indicators, especially T1 SI ratio < 0.73 for MRI and relative washout > 11.5 for CECT, can support physicians in diagnosing and treating patients effectively.

## Introduction

The incidental detection of localized solid renal masses has been rising continuously over the last few decades without an increase in mortality.^1^ This is due to the fact that a significant proportion of these tumors are benign or indolent and predominantly do not require treatment, as their size will increase very slowly or will not show any noticeable growth over time.^2^ The vast majority of them are small renal masses whose diameter does not exceed 4 cm. Accurate characterization of low-diameter lesions within such a heterogeneous group of possible pathologies has been notoriously difficult when relying on imaging features. In fact, data shows that approximately 16% of these indeterminate small solid renal masses which were resected turned out to be benign neoplasms, specifically, oncocytoma or angiomyolipoma (AML).^3,4^ Moreover, many histopathologically proven renal cancers show indolent behaviour and in some cases can be managed with an active surveillance.^5,6^ However, it should be noted that kidney cancer accounts for 3% of all malignancies, out of which the most common diagnosis is renal cell carcinoma with a mortality rate of 30–40%.^7^ Histological classification of this cancer differentiates it into three subtypes - clear cell, papillary and chromophobe, sequentially according to the frequency of their occurrence. These data indicate the need to improve presurgical characterization of renal masses such as imaging features and, as a result, minimize avoidable overtreatment.

**Figure 1.**
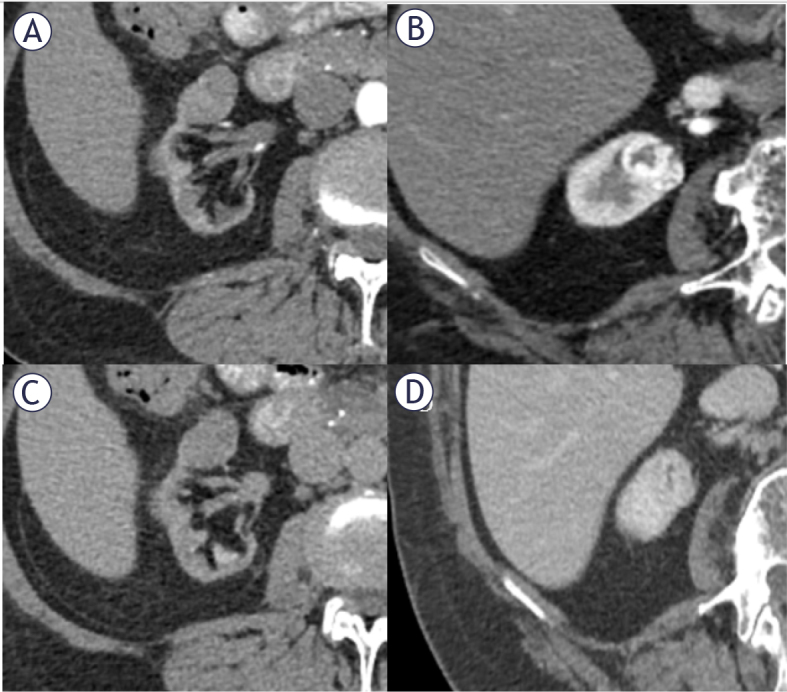
Axial CT images of two anteriorly localized kidney masses that were subsequently resected and histopathologically verified as benign **(A, C)** and malignant **(B, D)** in corticomedullary and nephrogenic phases, respectively.

The most typical modalities for imaging renal masses include ultrasound, contrast enhanced computer tomography (CECT), and magnetic resonance imaging (MRI). In many cases, ultrasound is a widely available, inexpensive, and harmless method of workup when a tumor is expected to be found. Therefore, despite the reduced sensitivity in detecting small masses, it is the preferred method of initial imaging in patients with contraindications to iodine contrast agents.^8^ However, CECT remains the method of choice for the assessment of renal tumors. It is also the gold standard for staging renal cell carcinoma regardless of the stage of advancement. The sensitivity of this method reaches 90% in tumors smaller than 2 cm in diameter and increases up to 100% in larger tumors.^9^ Non-enhanced, corticomedullary, and nephrographic phases are typical components of the standard “renal mass” protocol.^10^ Examples of benign and malignant solid renal masses in corticomedullary and nephrogenic phases are depicted in Figure 1.

Magnetic resonance imaging is an alternative to the above-mentioned methods and is particularly useful for distinguishing solid tumors from cystic masses, especially when the CECT result is inconclusive. Although standard MRI sequences have not been shown to be more sensitive than CECT in differentiating clear cell carcinoma from tumors such as oncocytoma, some studies have suggested that it is a promising method for distinguishing fat-poor AML from renal cell carcinoma (RCC).^11^ Another method is imaging-guided percutaneous biopsy, which is used for preoperative histopathological characterization of renal tumors. Since the development of immunohistochemistry, imaging-guided percutaneous biopsy is characterized by its high accuracy, estimated at up to 70-90%. However, it is one of the less frequently chosen techniques because of the associated risk and should be considered in masses which remain indeterminate after initial imaging.^12^ Moreover, newer modalities are currently being evaluated such as contrast-enhanced ultrasound and advanced applications of MRI.^8^

The aim of our study was to assess the performance of CECT in the characterization of renal masses, differentiating ccRCC from other renal masses on the base of quantitative imaging features. Additionally, we aimed to compare the performance of CECT and MRI in the characterization of small renal masses (SRMs) in selected aspects.

## Patients and methods

### Studied population

This retrospective, observational, single-center study was performed in the Department of Diagnostic Imaging in the University Hospital of Krakow and included 51 patients with indeterminate SRMs detected on CECT imaging. Final diagnoses were made on histopathologic results as standard reference (n = 38) or based on clinical and imaging features (diagnosis of lipid-poor angiomyolipoma or non-ccRCC masses without exact subtype information) if a regression or complete lack of progression was observed during follow-up (minimum 36 months). Forty-two of these patients also had available MRI results. The study was approved by the Ethics Committee and the requirement for patient informed consent was waived (OIL/KBL/51/2023).

### Computed tomography studies

All CECT examinations were performed using multirow helical scanners (64, 128, or 256 rows) and the following parameters: slice thickness 1.25–2.5 mm, tube voltage 120 kV, tube current-time product 50–250 mAs, matrix 512 × 512 pixels. Computed tomography images in non-enhanced, corticomedullary, nephrographic, and excretion phases were obtained. Non-ionic agents were injected at a rate of 3.5–4.0 ml/s with volume 450 mg I/kg of body weight. In solid lesions, the region of interest for attenuation measurement was positioned over the area that exhibited the greatest postcontrast enhancement. Computed tomography studies were assessed by two radiologists with at least 3 years of experience, blinded to the final diagnosis.

The assessed CECT features were categorized into four groups: (1) Anatomical, including the mass’s location in the right kidney or left kidney, determined by its position within the renal parenchyma; (2) Morphological, encompassing bean-like shape (an elongated, irregular contour resembling a bean), ball-like shape (a rounded, spherical appearance), and heterogeneous mass (non-uniform density indicating varied tissue components such as necrosis or viable tumor), all assessed qualitatively; (3) Enhancement, comprising significant enhancement (> 15 Hounsfield Units [HU], measured as the difference between pre- and post-contrast phases to reflect vascularity), heterogeneous enhancement (non-uniform contrast uptake across the mass), washout pattern (rapid contrast clearance, defined as a decrease of at least 20 HU between corticomedullary and nephrographic phases), prolonged pattern (sustained or slow contrast enhancement into the delayed phase), enhancement in corticomedullary phase (contrast uptake 30–60 seconds post-injection), difference in enhancement between corticomedullary and native phase (increase in HU from pre-contrast to corticomedullary phase), difference in enhancement between corticomedullary and nephrographic phase (HU change from corticomedullary to nephrographic phase, typically 70–120 seconds post-injection), absolute washout (calculated as [(HU corticomedullary – HU nephrographic) / (HU corticomedullary – HU native)] × 100, reflecting proportional contrast clearance), relative washout (calculated as [(HU corticomedullary – HU nephrographic) / HU corticomedullary] × 100, indicating percentage clearance from peak enhancement), difference in enhancement between nephrographic and native phase (HU increase from pre-contrast to nephrographic phase), and enhancement in nephrographic phase (contrast uptake in the nephrographic phase), all evaluated visually and quantitatively using region-of-interest measurements; and (4) Secondary Features, including tumor-feeding vessels (visible vascular structures supplying the mass, indicating angiogenesis), necrosis area (nonenhancing, low-density regions consistent with tissue death), and calcification (high-density calcium deposits within the mass), identified through visual inspection and density analysis.

### Magnetic resonance imaging studies

Magnetic resonance imaging studies were performed using 1.5T or 3.0T scanners and included all the necessary sequences for lesion assessment according to the ccLS (Clear Cell likelihood score) v2.0: axial and coronal 2D T2w single shot acquisitions, axial 2D T1w gradient echo in and out of phase for chemical shift imaging, pre- and (dynamic) postcontrast 3D T1w SPGR with fat saturation including delayed scans and diffusion-weighted imaging. Detailed information regarding our MRI protocol and analyzed MRI parameters was described previously.^13^ The MRI parameters assessed included: (1) Intense corticomedullary enhancement, defined as strong contrast uptake in the corticomedullary phase (typically 30–60 seconds post-gadolinium injection), reflecting robust vascularity; (2) Clear Cell Likelihood Score (ccLS) = 5, a qualitative score from a standardized system assessing the highest likelihood of ccRCC based on MRI features such as enhancement patterns, signal intensity, and heterogeneity, assigned by radiologists; and (3) ccLS = 4/5, a combined score reflecting high to very high likelihood of ccRCC, similarly derived from visual interpretation of MRI characteristics; (4) arterial-to-delayed ratio (ADER), obtained as the ratio of difference between signal intensity in the corticomedullary phase and pre-contrast images to the difference in signal intensity in delayed phase and pre-contrast images; and (5) T1 SI measured as the ratio of tumor SI to renal cortex SI. According to our previous results, we used the following cut-off point for the MRI parameters: ADER > 0.99 and T1 SI (signal intensity) ratio < 0.73.^13^

### Statistical analysis

The Student’s t-test or the Mann-Whitney test was used to compare continuous variables depicted as means ± standard deviations or medians and interquartile ranges (IQR). Pearson’s χ2 test or Fisher’s exact test was used to evaluate categorical variables, which are presented as numbers and percentages. Receiver operating characteristic (ROC) analysis was performed to calculate the area under the curve (AUC) and was used to determine which of the selected CECT features are discriminators between ccRCC and other types of SRMs. A logistic regression was performed in order to determine which of the CECT and MRI parameters are significant predictors of ccRCC. Specificity, sensitivity, positive predictive value (PPV), negative predictive value (NPV), accuracy, and negative likelihood ratio were calculated for each tested criterion. A p-value of 0.05 or less was considered statistically significant. Statistical analyses were performed with IBM SPSS Statistics (version 24, IBM Corp., Armonk, NY, USA). Confidence intervals (CI) were calculated using MEDCALC (free statistical calculators).

## Results

### Difference in symptoms and risk factors presented in patients with ccRCC *vs*. patients with other etiologies of indeterminate small renal masses

**Figure 2.**
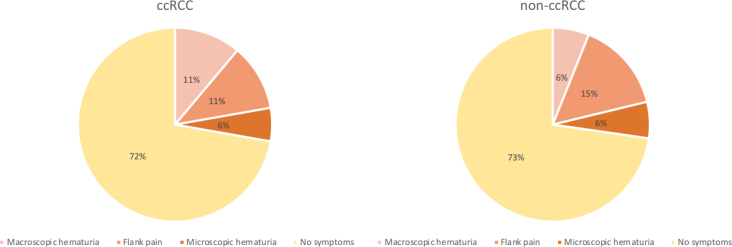
Prevalence of observed symptoms in patients with ccRCC and non-ccRCC. ccRCC = clear cell renal cell carcinoma; non-ccRCC = other than clear cell renal cell carcinoma

A total of 51 patients (49% male) with indeterminate solid SRMs were retrospectively enrolled in the study. In the majority of patients, symptoms indicating the presence of a renal mass were not present (Figure 2). Patients with ccRCC did not differ significantly from patients with other than ccRCC etiology of renal masses in terms of presented symptoms: macroscopic hematuria (2 [11.1%] *vs*. 2 [6.1%], p = 0.607), flank pain (2 [11.1%] *vs*. 5 [15.2%], p = 1), microscopic hematuria (1 [5.6%] *vs*. 2 [6.1%], p = 1).

The population was also examined for the presence of individual risk factors. Different risk factors were more commonly detected in the ccRCC group when compared to the non-ccRCC group (94.4% *vs*. 66.7%, p = 0.037). In the ccRCC group, there were significantly more smoking patients than in the non-RCC group (11 [61.1%] *vs*. 9 [27.3%], p = 0.018). Similarly, there were more hypertensive patients in the ccRCC group when compared to the non-ccRCC group (14 [77.8%] *vs*. 16 [48.5%], p = 0.042) (Table 1).

**Table 1.** Differences in risk factors detected in patients with ccRCC *vs*. patients with other etiologies of indeterminate small renal masses (SRMs)

Risk factors	ccRCC group (n = 18)	non-ccRCC group (n = 33)	p-value
All risk factors	17 (94.4%)	22 (66.7%)	0.037
Smoking	11 (61.1%)	9 (27.3%)	**0.018**
Family history of neoplasm	0 (0%)	1 (3%)	1
Genetic syndrome	0 (0%)	0 (0%)	1
Dialysis related cystic disease	0 (0%)	0 (0%)	1
Obesity	0 (0%)	3 (9.1%)	0.544
Hypertension	14 (77.8%)	16 (48.5%)	**0.042**
Cyclophosphamide treatment	1 (5.6%)	1 (3.0%)	1
Male sex	6 (33.3%)	19 (57.6%)	0.098

1 ccRCC = clear cell renal cell carcinoma; non-ccRCC = other than clear cell renal cell carcinoma.

### Significance of various CECT parameters in patients with ccRCC in comparison to patients with other etiologies of indeterminate small renal masses

Many of the compared CECT parameters differed significantly between the ccRCC group and the non-ccRCC group, as detailed in Table 2. Heterogenous mass (indicating non-uniformity in density due to varied tissue components such as necrosis or viable tumor) was observed significantly more often in the ccRCC group (88.9% *vs*. 60.6%, p = 0.034) compared to the non-ccRCC group, where homogenous mass dominated (11.1% *vs*. 39.4%, p = 0.034), suggesting greater structural complexity in ccRCC. Similarly, heterogeneous enhancement (where contrast uptake varies across the mass due to differing vascularity or tissue composition) was more frequent in the ccRCC group than the non-ccRCC group (83.3% *vs*. 51.5%, p = 0.025) where homogenous enhancement dominated (16.7% *vs*. 48.5%, p = 0.025), reflecting varied contrast uptake within ccRCC lesions. Moreover, presence of tumor-feeding vessels, identified as distinct vascular structures supplying the mass and reflecting increased angiogenesis, was also significantly greater in the ccRCC group (33.3% *vs*. 3.0%, p = 0.006), highlighting increased angiogenesis in ccRCC. Presence of a washout pattern, defined as a rapid decrease in enhancement (at least 20 HU difference between corticomedullary and nephrographic phases), indicative of quick contrast clearance, was more commonly observed in patients with ccRCC when compared to non-ccRCC etiology of SRMs (83.3% *vs*. 33.3%, p < 0.001). On the other hand, prolonged enhancement, characterized by sustained or slow contrast enhancement into the delayed phase, suggesting less rapid clearance, was detected more commonly in the non-ccRCC group when compared to the ccRCC group (66.7% *vs*. 16.7%), p < 0.001).

**Table 2.** Differences in CECT parameters in patients with ccRCC and patients with other etiologies of indeterminate small renal masses

CECT Features	ccRCC (n = 18)	Non-ccRCC (n = 33)	p-value
**Anatomical**			
Right kidney	55.6% (10)	36.4% (13)	0.268
Left kidney	44.4% (8)	60.6% (20)	0.268
**Morphological**			
Bean-like shape	38.9% (7)	27.3% (9)	0.393
Ball-like shape	61.1% (11)	72.2% (24)	0.393
Heterogeneous mass	88.9% (16)	60.6% (20)	0.034*
**Enhancement**			
Significant (> 15 HU)	94.4% (17)	81.8% (27)	0.398
Heterogeneous enhancement	83.3% (15)	51.5% (17)	0.025*
Washout pattern	83.3% (15)	33.3% (11)	< 0.001*
Prolonged pattern	16.7% (3)	66.7% (22)	< 0.001*
**Secondary Features**			
Tumor-feeding vessels	33.3% (6)	3.0% (1)	0.006*
Necrosis area	44.4% (8)	21.2% (7)	0.082
Calcification	5.6% (1)	9.1% (3)	1.000

1 CECT = contrast enhanced computed tomography; ccRCC = clear cell renal cell carcinoma; HU = Hounsfield Units; non-ccRCC = other than clear cell renal cell carcinoma.

The anatomical location of the mass in either the right or left kidney did not significantly differ between the ccRCC and non-ccRCC groups. Morphologically, masses were classified as either bean-like, with an elongated, irregular contour, or ball-like, with a rounded, spherical appearance, but these shape differences between the ccRCC and non-ccRCC groups were not significant (p = 0.393), suggesting morphology alone is not discriminative. Similarly, significant enhancement, defined as an increase in density greater than 15 Hounsfield Units (HU) post-contrast, reflecting substantial vascularity was not significant.

There were also no significant differences between the ccRCC and non-ccRCC groups in necrosis area (defined as non-enhancing, low-density regions within the mass consistent with tissue death), calcification (identified as high-density calcium deposits within the mass), thrombosis (characterized as a thrombus within associated vasculature, *e.g*., renal vein, appearing as a filling defect), or macroscopic fat (described as very low-density fat tissue within the mass), indicating these features are not distinguishing between the two etiologies.

**Figure 3.**
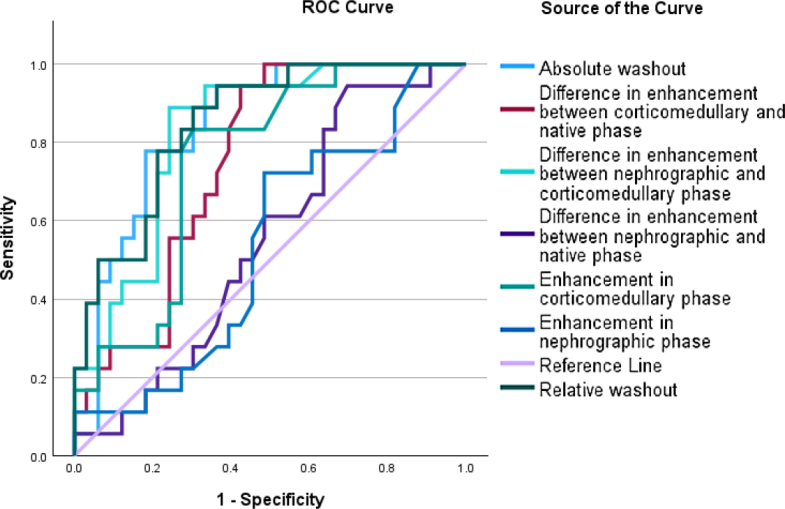
ROC analysis of the selected CT parameters.

Receiver operating curve analysis revealed that from the selected CECT features reflecting contrast enhancement, relative washout (difference in enhancement between corticomedullary and nephrographic phase / enhancement in corticomedullary phase)*100 had the highest AUC value (0.850) for the prediction of ccRCC occurrence (Table 3). Receiver operating curves are depicted on Figure 3.

**Table 3.** Area under the curve analyses according to selected CECT parameters in the prediction of ccRCC occurrence

CECT features	AUC	p-value
Enhancement in corticomedullary phase	0.752	**0.003**
Difference in enhancement between corticomedullary and native phase	0.751	**0.003**
Difference in enhancement between corticomedullary and nephrographic phase	0.828	**< 0.001**
Absolute washout - (difference in enhancement between corticomedullary and nephrographic phase/difference in enhancement between corticomedullary and native phase)*100	0.842	**< 0.001**
Relative washout - (difference in enhancement between corticomedullary and nephrographic phase/enhancement in corticomedullary phase)*100	0.850	**< 0.001**
Difference in enhancement between nephrographic and native phase	0.552	0.541
Enhancement in nephrographic phase	0.535	0.679

1 AUC = area under the curve; ccRCC = clear cell renal cell carcinoma; CECT = contrast enhanced computed tomography

### Clinical features and CECT imaging parameters in the prediction of ccRCC

Univariate logistic regression analysis demonstrated that smoking and different CECT parameters were predictors of ccRCC (Table 4). In multivariate analysis, only relative washout and smoking remained as significant predictors of ccRCC occurrence (OR: 1.19, 95% CI: 1.01–1.41, p = 0.042; OR: 7.50, 95% CI: 1.13–49.88, p = 0.04; Table 4).

**Table 4.** Clinical features and CECT parameters in the prediction of ccRCC occurrence in univariate and multivariate logistic regression analysis

Tested features	Univariate OR (CI: 95%), p-value	Multivariate OR (CI: 95%), p-value
**Clinical parameter**		
Smoking	4.19 (1.24–14.17), p = 0.02	7.50 (1.13–49.88), p = 0.04
Hypertension	2.92 (0.79–10.76), p = 0.11	-
**CECT parameter**		
Heterogeneous mass	5.2 (1.02–26.47), p = 0.05	-
Heterogeneous enhancement	4.71 (1.14–19.34), p = 0.03	4.51 (0.47–43.59), p = 0.19
Washout pattern (at least 20 HU between corticomedullary and nephrographic phases)	10.0 (2.38–42.01), p = 0.002	0.13 (0.01–3.28), p = 0.22
Relative washout - (difference in enhancement between corticomedullary and nephrographic phase/enhancement in corticomedullary phase)*100	1.08 (1.03–1.14), p = 0.001	1.19 (1.01–1.41), p = 0.04
Enhancement in corticomedullary phase	1.02 (1.00–1.03), p = 0.014	1.03 (0.95–1.12), p = 0.46
Difference in enhancement between corticomedullary and native phase	1.02 (1.00–1.03), p = 0.019	0.98 (0.91–1.06), p = 0.62
Difference in enhancement between nephrographic and corticomedullary phase	0.95 (0.92–0.98), p = 0.002	1.05 (0.95–1.16), p = 0.35

1 ccRCC = clear cell renal cell carcinoma; CECT = contrast enhanced computed tomography; CI = confidence interval; OR = odds ratio

### New approaches in the characterization of intermediate small renal lesions

**Figure 4.**
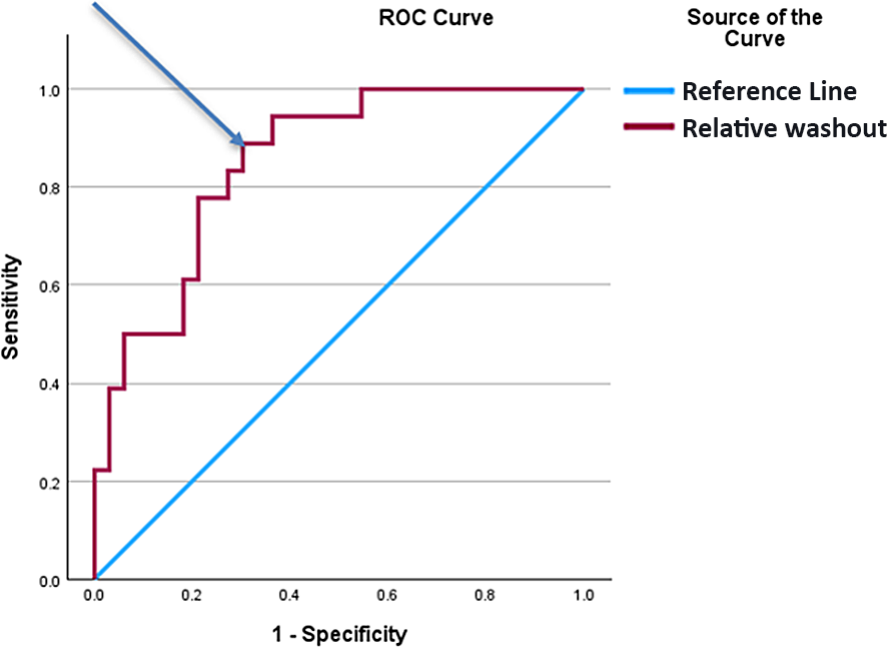
The cut-off point is determined in the ROC analysis using the Youden index. Youden index = 0.59; Suggested cut-off: 11.54

Using the Youden index, we determined the proposed cut-off point for relative washout in the ROC analysis to be 11.54 (Figure 4). Then, we compared the relative washout parameter with the newly established cut-off value with selected novel MRI score/parameters (Table 5). In results we found that only T1 SI ratio < 0.73 (defined as the lesion’s signal intensity relative to the renal cortex on precontrast T1-weighted MRI), ccLS = 5 (a qualitative score indicating the highest likelihood of ccRCC based on MRI features like enhancement and heterogeneity), and relative washout > 11.5 (calculated as the percentage decrease in HU from peak to delayed phase on CECT reflecting rapid contrast clearance) were different in patients with ccRCC when compared to the non-ccRCC group (27.8% *vs*. 3.0%, p = 0.007; 16.7% *vs*. 0.0%, p = 0.024; 88.9% *vs*. 30.3%, p < 0.001; Table 5). The parameters that did not significantly differ between the ccRCC and non-ccRCC groups included intense corticomedullary enhancement (defined as strong contrast uptake in the corticomedullary phase of MRI), the ADER > 0.99 (a diffusion-weighted imaging metric comparing Apparent Diffusion Coefficient before and after contrast), and ccLS = 4/5 (a combined score reflecting high to very high likelihood of ccRCC).

**Table 5.** Difference in selected novel MRI and CECT parameters between ccRCC and non-ccRCC

Imaging features	ccRCC group (n = 12)	non-ccRCC group (n = 30)	p-value
**MRI parameters**			
Intense corticomedullary enhancement	6 (50.0%)	14 (46.7%)	0.529
ADER > 0.99	9 (75.0%)	14 (46.7%)	0.291
T1 SI ratio <0.73	5 (41.7%)	1 (3.3%)	**0.007**
ccLS = 5	3 (25.0%)	0 (0.0%)	**0.024**
ccLS = 4/5	6 (50.0%)	11 (36.7%)	0.590
**CECT parameter**			
Relative washout > 11.5	12 (100.0%)	10 (33.3%)	**< 0.001**

1 ADER = arterial to delayed enhancement ratio; ccLS = clear cell likelihood score; ccRCC = clear cell renal cell carcinoma; CECT = contrast enhanced computed tomography; non-ccRCC = other than clear cell renal cell carcinoma

In the next step, we put these MRI variables, relative washout with the new cut-off point, and smoking into multivariable analysis. We observed that only T1 < 0.73 and relative washout >11.5 were independent predictors of ccRCC occurrence (OR: 30.86, 95% CI: 1.58–600.26, p = 0.024; OR: 15.36, 95% CI: 1.52–155.16, p = 0.021). When we compared CECT and MRI parameters, we noticed that the best accuracy (76.5%) and sensitivity (88.9%) were observed for relative washout, with a good specificity of 69.7%. Table 6 shows specificity, sensitivity, accuracy, positive likelihood ratio, and positive predictive value for these selected imaging parameters.

**Table 6.** Specificity, sensitivity, accuracy, positive likelihood ratio, and positive predictive value for selected imaging parameters

Imaging feature	Sensitivity	Specificity	Accuracy	PLR	PPV
**MRI parameter**					
Intense CM phase enhancement	50.0 (21.1–78.9)%	53.3 (34.3–71.7)%	52.4 (36.4–68.0)%	1.1 (0.5–2.1)	30.0 (17.8–45.9)%
ADER > 0.99	75.0 (42.8–94.5)%	53.3 (34.3–71.7)%	59.5 (43.3–74.4)%	1.6 (1.0–2.7)	39.1 (28.0–51.5)%
T1 SI ratio < 0.73	41.7 (15.2–72.3)%	96.7 (82.8–99.9)%	81.0 (65.9–91.4)%	12.5 (1.6–96.1)	83.3 (39.4–97.5)%
ccLS = 5	25.0 (5.5–57.2)%	100.0 (88.4–100.0)%	78.6 (63.2–89.7)%	-	100.0 (29.24–100.0)%
ccLS = 4/5	50.0 (21.1–78.9)%	63.3 (43.9–80.1)%	59.5 (43.3–74.4)%	1.4 (0.7–2.9)	35.3 (20.7–53.2)%
**CECT parameter**					
Relative washout > 11.5	100.0 (73.5–100.0)%	66.7 (47.2–82.7)%	76.2 (60.6–88.0)%	3.0 (1.8–5.0)	54.6 (42.0–66.6)%

1 ADER = arterial to delayed enhancement ratio; ccLS = clear cell likelihood score; CM = corticomedullary; PLR = positive likelihood ratio; PPV = positive predictive value

## Discussion

Masses measuring > 70 HU in the non-enhanced phase are mostly benign if they do not show features typical for malignancy such as calcifications, thickened walls or multiple septations. The best example confirming the above statement is a hemorrhagic cyst, which is the diagnosis in up to 99.9% of cases of a homogenous mass with high HU.^14^ At the same time, it is the least frequently observed range of values in imaged renal masses. Most researchers agree that tumors with an attenuation between 20 and 70 HU are indeterminate and require further diagnostics. The vast majority of masses with attenuation < 20 HU are benign and do not need detailed evaluation; however, Badri *et al*. demonstrated that this group comprises 50% of all papillary RCC, with a significant predominance of type 2 with possibly worse prognosis.^15,16^ According to Abdelmegeed *et al*., high pre-contrast attenuation of 41.7 ± 6.823 HU is observed in Xp11 translocation RCC and it is the apical value observed among renal tumors. Slightly lower values that oscillate in the range of 38.18 ± 4.36 HU are noted in lipid-poor AML, while values of 35.47 ± 4.23 HU are seen in papillary RCC.^17^ O’Connor *et al*. proposed that RCC, confirmed later in followup, rarely show an HU beyond the limit of 20-70 on non-enhanced CT, while Krishna *et al*. suggested that RCC measuring < 20 HU may simulate renal cyst on non-enhanced CECT despite their heterogeneous structure and irregular margins.^18,19^

Our results indicate that heterogeneous mass and heterogeneous enhancement are significantly more common in the ccRCC group (88.9% and 83.3%) compared to the non-ccRCC group, wherehomogeneous lesions predominated (11.1% and 16.7%). The greater frequency of heterogeneity may suggest a more aggressive nature of the tumor, which is confirmed in the literature. Studies have noted that ccRCC often shows greater structural complexity and differences in blood supply, which may be related to the angiogenesis process typical of tumors. Tumors with the greatest corticomedullary phase enhancement include ccRCC and oncocytomas. According to Zhang *et al*., there are two more groups.^20^ The first group includes intermediate-enhancing tumors such as chromophobe carcinomas and fat-poor AML, while the second group comprises mostly hypovascular tumors and consist of papillary RCC. Differentiation between ccRCC and oncocytoma can be difficult even despite the existence of the central stellate scar in the latter, as it is present only in up to 33% of them.^21^ Moreover, Campbell *et al*. stated that a scar may be present in a small fraction of RCCs, and additionally, areas of necrosis occurring in RCC can imitate it.^22^ Hence, the most promising method of distinguishing these tumors seems to be their characteristic contrast enhancement and selected MRI parameters. The most common pattern for ccRCC is strong enhancement in the corticomedullary phase, intermediate to high washout, high signal intensity on T2-weighted image, and signal drop on opposed phase gradient echo image.^23^ Despite having the same enhancement pattern, oncocytoma typically peaks later in the nephrographic phase.^24^

We observed a significant difference in the presence of tumor-feeding vessels between the two groups (33.3% in ccRCC *vs*. 3.0% in non-ccRCC) and a greater prevalence of the washout pattern in the ccRCC group (83.3% *vs*. 33.3%), which emphasizes the importance of angiogenic parameters in the diagnostic process. This association suggests that CECT may be a valuable tool in risk assessment of SRMs.^25^ The surprising results of prolonged enhancement in the non-ccRCC group (66.7%) compared to the ccRCC group (16.7%) indicate a different pathological mechanism in this group. Prolonged enhancement may indicate inflammatory or cystic processes, which are more common in benign renal lesions.^14^ In a study conducted on 163 patients, Kopp *et al*. showed that a washout value ≥ 0 indicates ccRCC, oncocytoma, and AML with a sensitivity and NPV of 100%. Moreover, the diagnosis of clear cell carcinoma in this case has a specificity of 35.2% and PPV of 66.7%.^26,27^ On MRI, ccRCC shows rapid washout in later phases, which results in it becoming hypointense to the renal cortex by the excretory phase.^27^ Bird *et al*. demonstrated that arterial phase enhancement > 500% and washout > 50% are specific features allowing the diagnosis of oncocytoma.^28^ Xie *et al*. stated that lipid-poor AML shows lower washout value in comparison with ccRCC and is 35.8 ± 32.7 HU *vs*. 48.3 ± 28.4 HU.^29^

Our results showed that only T1 SI ratio < 0.73 on MRI and relative washout > 11.5 from CECT remain independent predictors of ccRCC (OR: 30.86, 95% CI: 1.5–600.26, p = 0.024; OR: 15.36, 95% CI: 1.52–155.16, p = 0.021). This reflects tumor angiogenesis characteristic for ccRCC, which is confirmed in the literature. The association between these indicators and the presence of ccRCC emphasizes the role of these parameters in assessing the risk of renal tumor.^30^ These values suggest that the use of both CECT and MRI indicators in clinical practice can support physicians in making decisions regarding the diagnosis and treatment of ccRCC patients.^2^ Computed tomography has been shown to have a greater sensitivity in detecting SRMs compared to MRI in many comparative studies.^23,31^ Computed tomography is also more effective in identifying regional changes, such as bleeding or calcification, and in assessing blood vessels in the context of malignant disease. However, MRI is often superior to CECT in assessing soft tissue characteristics and may offer better information on whether the tumor is benign or malignant.^23^ Studies have shown that in the diagnosis of specific types of renal tumors such as ccRCC, CECT may be more effective in identifying radiological features characteristic for this tumor, whereas MRI may be better at assessing soft tissue features and angioarchitecture of the tumor.

In our study, we noted that patients with ccRCC had a significantly greater percentage of risk factors, such as smoking and hypertension when compared to patients with other etiologies of renal tumors. According to recent literature, these factors are considered to be important elements influencing the development of renal cancer.^32^ Studies emphasize the importance of lifestyle and comorbidities in the pathogenesis of ccRCC, which suggests the need for intensive control of these factors in high-risk populations. Additionally, the lack of significant clinical symptoms in patients with indeterminate renal masses supports the thesis that early diagnosis and monitoring of patients with the possibility of developing ccRCC should be based on the identification of risky behaviours and diseases, and not only on the assessment of clinical symptoms.

## Conclusions

In clinical practice, the use of both CECT and MRI indicators, especially T1 SI ratio < 0.73 on MRI and relative washout > 11.5 on CT, can support physicians in making decisions regarding the diagnosis and treatment of patients.
